# Electrically tunable metasurface based on Mie-type dielectric resonators

**DOI:** 10.1038/srep43026

**Published:** 2017-02-21

**Authors:** Zhaoxian Su, Qian Zhao, Kun Song, Xiaopeng Zhao, Jianbo Yin

**Affiliations:** 1 Smart Materials Laboratory, Department of Applied Physics, Northwestern Polytechnical University, Xi’an, 710129, China; 2 State Key Laboratory of Tribology, Department of Mechanical Engineering, Tsinghua University, Beijing, 100084, China

## Abstract

In this paper, we have designed a metasurface based on electrically tunable Mie-type resonators and theoretically demonstrated its tunable response to electromagnetic waves with varying frequency. The metasurface consists of disk-like ferroelectric resonators arrayed on a metal film and the upper surface of resonators is covered by ion gel film which is transparent for incident electromagnetic wave. Using the metal film and ion gel film as electrodes, the permittivity of the resonators can be adjusted by an external electric field and, as a result, the reflection phase of the resonators can be dynamically adjusted in a relatively wide range. By programmable controlling the electric field strength applied on resonators of metasurface, a 2π phase ramp can be realized and, thereby, the arbitrary reflection behavior of incident waves with varied frequency is obtained. Because of the tunability, this metasurface can also be used to design adaptive metasurface lens and carpet cloak.

Metamaterial has attracted many interests in optics community because of its ability of manipulation of electromagnetic wave^1^. Along with the demand for integration and miniaturization of optical components, metasurface appears. Metasurface is a kind of special two-dimensional metamaterial with subwavelength structure periodicity and can tailor wave front according to people’s will. Compared to bulk metamaterial, metasurface has lower loss and it is easier to fabricate. Up to now, many kinds of metasurfaces have been designed and fabricated for different functions, such as steering^2^, focusing^3^, total absorption^4^, and so on. However, most of these metasurfaces are fabricated based on metallic resonators, which still have great inherent losses. What’s more, the phase shift generated by the units on the metasurfaces is fixed. As a result, the reflection or refraction angle of incident wave is fixed. Furthermore, the response bandwidth of most of the metasurfaces is also narrow. Although some broadband metasurfaces have been fabricated by coupling different size of resonator arrays, their efficiency is inevitably reduced. In addition, the metasurfaces are composed of resonators with different geometric dimensions for providing designed local phase shift, also leading to great difficulties in fabrication of large scale. All of these disadvantages limit the application of metasurface.

Recently, some kinds of metamaterials based on Mie-type dielectric resonators have been proposed^5^,^6^,^7^,^8^,^9^,^10^. The Mie resonance of dielectric resonators provides a novel way to create magnetic or electric resonance by displacement currents. Thus, the metamaterials based on Mie-type dielectric resonators show much lower losses compared to the metamaterials based on metallic resonators^11^. For example, Zou *et al*. have fabricated a metasurface based on dielectric nano-antennas for deflecting reflected wave at visible frequencies. Both simulation and measurement reveal that ~35% of the incident wave power can be reflected by the metasurface^12^. Cheng *et al*. have designed a dielectric metasurface that has a beam-tilting array and a large-scale lens function at 195 TH^10^. However, the shortcomings involving narrow bandwidth and lack of tenability still need to be effectively solved in the dielectric metasurfaces.

In this work, we have proposed a metasurface based on electrically tunable Mie-type dielectric resonators, which has a tunable response to electromagnetic wave with varying frequency. The metasurface consists of disk-like barium strontium titanate (BST) ferroelectric resonators arrayed on a metal film and the upper surface of resonators is covered by ion gel film. When an electric field is applied to the bottom metal film and upper ion gel film, the ion gel can be polarized due to ion motion and thus induce a largely local electric field^13^,^14^. This local electric field can facilely lead to the change of permittivity of the BST ferroelectric resonators. Thus, the resonators can provide a wide range of change in the reflection phase. By controlling the applied electric field strength on the resonator, we can adjust the reflection phase of a resonator to achieve the coverage of phase shift of 2**π**. We can also easily realize different phase gradient along the metasurface by applying different external electric field on the BST resonators. As a result, the arbitrary reflection angle of incident electromagnetic wave with varied frequency can be realized. We calculate the reflection phase as a function of the permittivity of resonators and simulate the realization of arbitrary reflection angle of incident wave with varied frequency. Finally, we use the tunable metasurface to design adaptive metasurface lens and carpet cloak, and demonstrate their wide-angle and tunable frequency characters by programmable control of electric field strength.

## Results and Discussion

### Mie resonance of dielectric resonator

**Figure 1:**
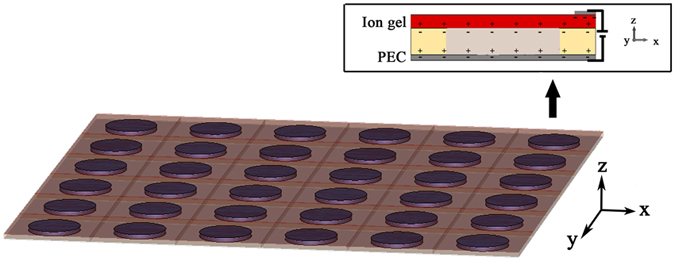
Scheme of proposed tunable metasurface. The permittivity of BST resonators in a line can be adjusted by external electric field.

Figure 1 shows the scheme of the proposed metasurface. It is composed of disk-like BST ferroelectric resonators arrayed on a metal film which can eliminate transmission. Here, the metal film is regarded as a perfect electric conductor (PEC). The diameter of each BST disk is 6.0 mm and the height is 0.5 mm. The periodicity of arrayed resonators is 10 mm. All of the dielectric disks are imbedded in a PMMA layer, which is sandwiched by a metal film and an ion gel film. The thickness of the ion gel film is 0.1 mm. The ion gel is a kind of mixture of ion liquid and polymer, which is transparent for incident electromagnetic wave because the ionic mobility is significantly slower compared to the frequency of microwave^15^,^16^. When an electric field is applied to the bottom metal film and upper ion gel film, the ion gel can be polarized due to ion motion and produce a large locally electric field (see Fig. 1). This large electric field can induce the change of permittivity (*ε*) of BST disks. According to the Mie theory, the resonance depends on the permittivity of dielectric resonators^17^. Therefore, by adjusting the permittivity of BST resonators, we can tune the resonance frequency and also the phase shift around the frequency of the Mie resonance. By programmable controlling the electric field strength applied on resonators, we can achieve the coverage of phase shift of 2**π** or wanted phase gradient to realize the arbitrary reflection angle of incident wave with varied frequency.

**Figure 2:**
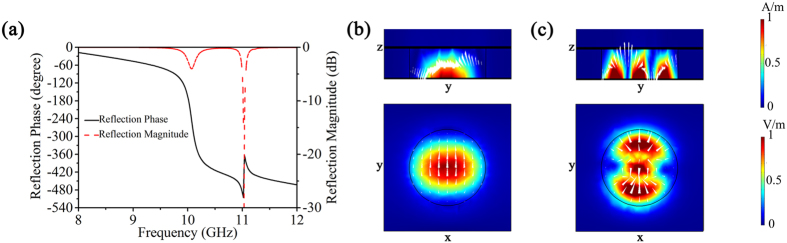
(**a**) The incident frequency dependence of reflection phase (solid line) and reflection magnitude (dash line) of one resonator unit cell. (**b**) The distribution of magnetic field intensity (top view) and electric field intensity (bottom view) in one resonator unit cell at 10 GHz. (**c**) The distribution of magnetic field intensity (top view) and electric field intensity (bottom view) in one resonator unit cell at 11 GHz. The direction and size of the white arrows in Fig. 2(b) and (c) represent the displacement current.

We firstly investigate the electromagnetic response of a unit cell containing a BST resonator. Figure 2(a) shows the simulation result of the magnitude and phase of reflection wave of the unit cell. We can find that the resonator exhibits two Mie resonances at about 10 GHz and 11 GHz, respectively. The corresponding distributions of normalized electric field intensity [bottom views (x-y plane)] and magnetic field intensity [top views (y-z plane)] at 10 GHz and 11 GHz are shown in Fig. 2(b) and (c), respectively. The white arrows in Fig. 2(b) and (c) represent the displacement current. From the top view of Fig. 2(b), we can see a strong magnetic field distribution in the disk, companied with circulating displacement current around the disk. It indicates that the first Mie resonance at 10 GHz is excited by the magnetic dipole resonance. From the top view of Fig. 2(c), however, we can see a strong field distribution in the disk, simultaneously companied with circulating displacement current and paralleled displacement current. It indicates that the second Mie resonance at 11 GHz is excited by the interaction of electric dipole resonance and magnetic dipole resonance. Both resonances have a wide range of phase shifts available at nearby frequencies. Compared to single magnetic dipole resonance, however, the interaction between electric and magnetic dipole resonances causes much larger loss as shown in Fig. 2(a). Therefore, we can employ the first Mie resonance at 10 GHz for the design of Mie-type smart metasurface.

### Electrically tunable metasurface

**Figure 3:**
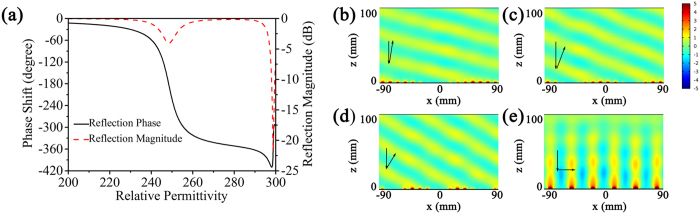
(**a**) The dependence of phase shift (solid line) and reflection magnitude (dash line) on the permittivity of BST resonators at 10 GHz. (**b**–**e**) The distribution of scattered electric field with different phase increment: (**b**) π/12, (**c**) π/4, (**d**) π/6, (**e**) π/3.

Under electric fields, the permittivity of BST resonators can be adjusted. Here, we make the permittivity of BST resonators be tuned from 200 to 300 with loss tangent of 0.004 when an external electric field is applied^18^. Figure 3(a) shows the calculated reflection phase shift at 10 GHz as a function of the permittivity of resonators. It can be found that the reflection phase shift can well cover a range of 0–2π, while the magnitude variation is small. Thus, we can periodically array the resonators and apply different external electric field strength to each array of BST resonators to produce a continuous phase shift covering full 2**π** and, thus, obtain the final smart metasurface. When a wave is normally incident to the metasurface, the reflected angle *θ* can be calculated by the following Eq. (1):


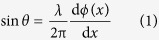


where *ϕ*(*x*) is the phase shift, *λ*_0_ is the free-space wavelength. It is noted that the reflected angle *θ* is determined by the phase increment d*ϕ*(*x*)/d*x*, which can be obtained by BST resonators with different permittivity. Thus, the reflected angle *θ* can be adjusted by changing the permittivity of the dielectric disks, i.e., by applying different external electric field on each array of resonators. We simulated the scattered electric field of the infinite array of sub-arrays with different phase increments in a unit length (π/12, π/4, π/6 and π/3) by changing the permittivity of the BST resonators. As shown in Fig. 3(b–e), the reflected angles with different phase increments are consistent with the theoretical values calculated from Eq. (1). In particular, it is interesting that the reflected wave will propagate along the surface with reflected angle 90° when the phase increment per unit length satisfies d*ϕ*(*x*) = π/3. Thus, we can transform the reflected wave into a surface wave.

**Figure 4:**
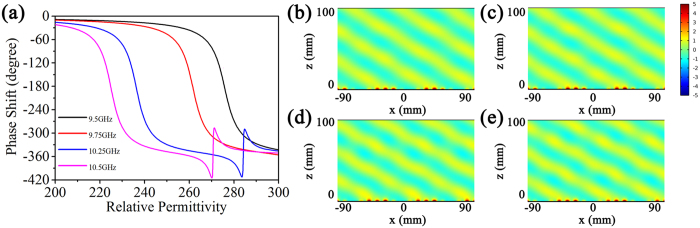
(**a**) The dependence of phase shift on the permittivity of the BST resonators at different incident frequency. (**b**–**e**) The distribution of scattered electric field at different incident frequency: (**b**) 9.5 GHz, (**c**) 9.75 GHz, (**d**) 10.25 GHz, (**e**)10.5 GHz.

Due to the dependence of resonances on the permittivity of BST resonators, we can also obtain a metasurface that can respond to incident electromagnetic waves with varied frequency. Figure 4(a) shows the calculated phase shift and reflection magnitude at different incident electromagnetic wave frequency. We can find that the phase shift still well covers the range of 0–2π or achieves a 2π phase ramp even if the incident frequency is different. Figure 4(b–e) show the simulated reflected field at different incident frequency when the permittivity of BST resonators is adjusted to provide a typical phase shift of π/6. It clearly shows that the off-normal reflection of light can be realized as soon as the dielectric disk possesses an appropriate permittivity to provide an appropriate local reflection phase.

### Electric field-dependent characteristic

**Figure 5:**
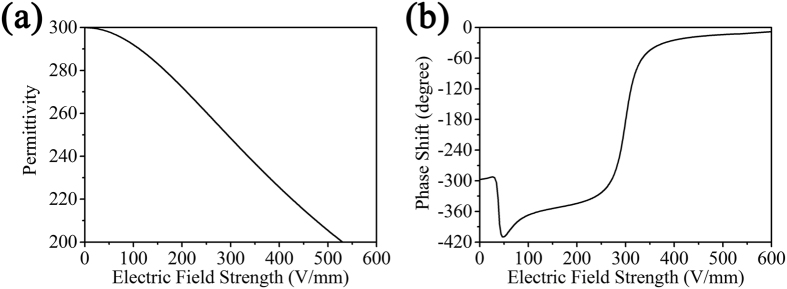
(**a**) The permittivity of the BST resonators as a function of electric field strength. (**b**) The phase shift of the BST resonators under different electric field strength.

The dependence of the permittivity (*ε*) of BST on a DC electric field strength (*E*_0_) at constant temperature can be written as^19^:


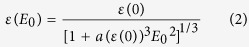


where *a* is phenomenological coefficient. We assume that the phenomenological coefficient *a* here is 3.13×10^−19^ m^2^V^−2^, which is as the same as the value derived in the previous work^19^. Thus we can estimate the permittivity of the BST resonator under different electric field strength, which is shown in Fig. 5(a). According to the dependence of the reflected phase shift on the permittivity of resonators, we can obtain the relation between phase shift and external electric field strength as shown in Fig. 5(b). When an external electric field is applied to upper ion gel film and bottom metal film, the ion gel will generate anion/cation separation as shown in Fig. 1, and the ions with opposite charge to another electrode will gather on the bottom surface of ion gel film, which leads to the most of the external electric field applying to the BST resonators. The thickness of BST resonators is 0.5 mm, thus, the electric field strength can be estimated according to Eq. (2). The estimated maximum voltage applied to the BST resonators to produce the wanted phase shift is 300 V. In the realistic fabrication, we need to use multi power supplies to apply different electric field on different array of resonators along y direction and, thus, we can realize 1D control of metasurface.

### Applications based on the smart metasurface

Based on the analysis above, through adjusting the permittivity of the BST resonators with an external electric field applied on each array of unit cells, the local phase shift of each array of unit cells can be adjusted. The response frequency and reflected angle of scattered wave of the metasurface can be arbitrarily adjusted without changing the structure of the metasurface, but only with the change of external electric field. Thus, the local phase response of the metasurface can be programed by changing the electric field applied on each array of unit cells. As a result, a metasurface which can be programed to achieve wanted wavefront is realized. This unique character makes the tunable metasuface possess potential applications in developing adaptive metasurface-based devices. In the following section, we further present two potential applications of the proposed tunable metasurface.

**Figure 6:**
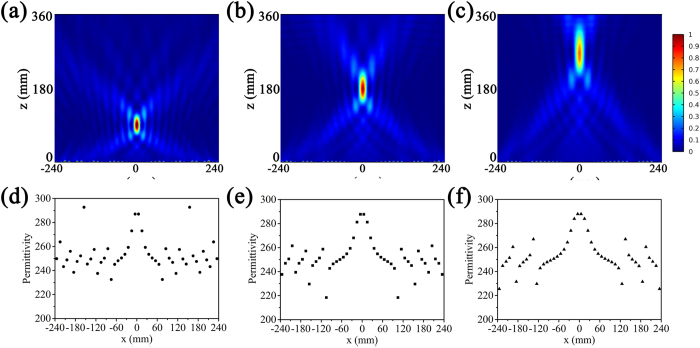
The distribution of the scattered field intensity |*E*|^2^ for the metasurface lens (**a**–**c**) and the permittivity of each array of resonators along x axis (**d**–**f**) with different focus location: *f* = 3*λ* (**a** and **d**), *f* = 6*λ* (**b** and **e**), *f* = 9*λ* (**c** and **f**).

The first example is that the proposed metasurface can be used to design adaptive metasurface lens. By adjusting permittivity of each array of BST resonator unit cells, we can control the phase distribution of metasurface, leading to different wavefront manipulations. For example, we can transform the metasurface into a flat lens to focus reflected wave when the phase shift along the metasurface follows the Eq. (3):


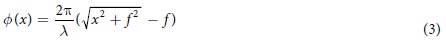


where *f* is the location of focus. From Eq. (3), we can obtain the phase shift *ϕ*(*x*) along x direction. According to the dependence of phase shift on the permittivity of the BST resonators shown in Fig. 2(a), the permittivity of the BST resonators along x direction at 10 GHz can be derived. When the permittivity of the BST resonators is set properly by the external electric field, the wanted phase profile described by Eq. (3) can be obtained. Thus, we can realize focus of the reflected wave with designed focus location as shown in Fig. 6(a–c). Figure 6(d–f) are the distribution of the permittivity of resonators along x axis corresponding to the Fig. 6(a–c). By applying external electric field on each array of unit cells, the permittivity of resonators will change along x axis. As soon as the permittivity of each resonator array along x axis satisfies the designed distribution, we can obtain the wanted phase profile. When further changing the electric field strength, the distribution of the permittivity of resonator arrays changes correspondingly and this leads to the change of local phase shift of resonators, and the focus location also can be changed. Thus, the metasurface can be used as an adaptive lens.

**Figure 7:**
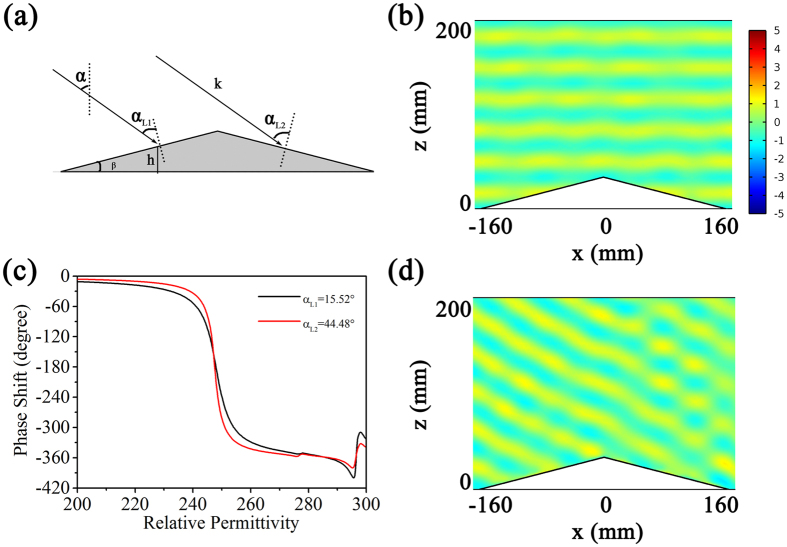
(**a**) Scheme of the triangular bump on the PEC ground. (**b**) The scattered field distribution in the case of normal incidence. (**c**) Phase shift of the unit cell for different local incident angles dependent on the permittivity of the BST resonators. (**d**) Scattered field distribution at incident angle *α* = 30°).

Because of the tunability of the permittivity of BST resonators, the metasurface can also be used to design active carpet cloak^20^,^21^. We simulated the carpet cloak based on the metasuface fabricated on a triangular bump. The scheme of the cloak and the triangular bump on the PEC ground is shown in Fig. 7(a). The height and the tilt angle *β* of the bump are 30 mm and sin^−1^ 0.25, respectively. To reconstruct the scattered field distribution, the phase shift at each point of the bump edge is calculated from Eq. (4).


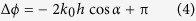


where *k*_0_ is the free space wave vector, *h* is the height of the point from the ground plane, and *α* is the incident angle of the incoming wave with respect to the ground plane. By adjusting the permittviy of BST resonators, the metasurface can provide a proper phase profile to make a phase compensation. Thus, the scattered field distribution around the bump is identical to the case that no bump is presented when the wave illuminates on the bump with the carpet cloak based on the metasurface. Due to the geometry of the triangular bump, the local incident angles of the incident wave are *α*_L1_ = *α*–*β* and *α*_L2_ = *α* + *β* for the left side and right side of the bump, respectively. In the case of normal incidence, we can neglect the influence of the local incident angle on the phase shift of the BST resonator unit cell, because the tilt angle *β* is small. According to the dependence of phase shift on permittivity shown in Fig. 5, we simulated the scatter field distribution in the case of normal incidence and the result is shown in Fig. 7(b). It is seen that the reflected wave is just like that of the electromagnetic wave reflected from a plane mirror at ground. In the case of oblique incidence, we should consider the influence of local incident angle *α*_L_ on the phase shift. The tilt angle of the triangular bump in our simulation is *β* = 14.48°. Figure 7(c) shows the dependence of phase shift on the permittivity of the BST resonators when the incident angle *α* = 30°, i.e., the local incident angles *α*_L1_ and *α*_L2_ are 15.52° and 44.48° respectively. The simulated result of scattered field is shown in Fig. 7(d). Similarly, it is seen that the reflected field distribution is identical to that reflected by a plane mirror at ground. These results illustrates that the metasurface cloak can be adjusted according to the incident angle.

## Conclusion

In conclusion, we have proposed an electrically tunable metasurface with adjustable response frequency based on the Mie-type resonance of the ferroelectric BST resonators. The ion gel is proposed to be used as upper electrode because it is transparent for incident electromagnetic wave at microwave frequency. We numerically investigate the resonance property of the BST resonators. Through applying an external electric field, the permittivity of the BST resonators can be adjusted. As a result, the local phase shift can be adjusted. Different reflected angle can be achieved by adjusting the permittivity of the BST resonators. Meanwhile, the response frequency of the metasurface also can be adjusted from 9.5 GHz to 10.5 GHz. The metasurface can realize different wavefront manipulations. It can be transformed into a flat lens to focus reflected wave, and its focus distance can be adjusted by changing the permittivity of BST resonators. Through adjusting the phase profile of the metasurface, a tunable carpet cloak according to different incident angles can be realized. The metasurface cloak also has the potential of tunable response frequency. The presented metasurface is easy to fabricate and is a good candidate for active or adaptive optical devices.

## Methods

In this paper, we use Comsol Multiphysics based on finite element method to calculated the reflection and field distribution. In the simulation of reflection dependent on incident frequency, the permittivity of BST resonators is set as 245, and its loss tangent is set as 0.004^22^. The permittivity of ion gel is set as 4 + 0.1*i*^16^. And the magnetic field component of the incident wave is along y axis, set as **H** = **y**exp[i(*k*_*x*_*x* + *k*_*z*_*z*)]. Perfectly matched layers are utilized along x axis to eliminate the reflected waves by the outer boundaries.

## Additional Information

**How to cite this article**: Su, Z. *et al*. Electrically tunable metasurface based on Mie-type dielectric resonators. *Sci. Rep.* **7**, 43026; doi: 10.1038/srep43026 (2017).

**Publisher's note:** Springer Nature remains neutral with regard to jurisdictional claims in published maps and institutional affiliations.
